# Supplementary material for: Multi‐dataset identification of innovative feature genes and molecular mechanisms in keratoconus
Source: J Cell Mol Med. 2024 Sep 19;28(18):e70079. doi: 10.1111/jcmm.70079 (PMC11412914; doi:10.1111/jcmm.70079)
Supplement: Supplementary file 2 — Material S2. [file JCMM-28-e70079-s002.zip › 7.GSEA analysis/1.ARL11/KEGG_GAP_JUNCTION.html]

Details for gene set KEGG\_GAP\_JUNCTION[GSEA]

|  || Dataset | ARL11.group.cls#h\_versus\_l |
| Phenotype | group.cls#h\_versus\_l |
| Upregulated in class | h |
| GeneSet | KEGG\_GAP\_JUNCTION |
| Enrichment Score (ES) | 0.18264209 |
| Normalized Enrichment Score (NES) | 0.82649404 |
| Nominal p-value | 0.87349397 |
| FDR q-value | 1.0 |
| FWER p-Value | 1.0 |
Table: GSEA Results Summary

  

Fig 1: Enrichment plot: KEGG\_GAP\_JUNCTION      
 Profile of the Running ES Score & Positions of GeneSet Members on the Rank Ordered List

  

| SYMBOL | TITLE | RANK IN GENE LIST | RANK METRIC SCORE | RUNNING ES | CORE ENRICHMENT || 1 | PLCB2 | na | 134 | 0.619 | 0.0664 | Yes |
| 2 | TUBAL3 | na | 1370 | 0.351 | 0.0705 | Yes |
| 3 | GRM5 | na | 2391 | 0.271 | 0.0718 | Yes |
| 4 | TUBB4A | na | 3479 | 0.220 | 0.0653 | Yes |
| 5 | GJD2 | na | 3894 | 0.204 | 0.0765 | Yes |
| 6 | TUBB8 | na | 5057 | 0.164 | 0.0616 | Yes |
| 7 | ADCY3 | na | 5276 | 0.158 | 0.0732 | Yes |
| 8 | ADCY2 | na | 5416 | 0.154 | 0.0867 | Yes |
| 9 | ADCY9 | na | 5439 | 0.153 | 0.1035 | Yes |
| 10 | LPAR1 | na | 5529 | 0.151 | 0.1180 | Yes |
| 11 | ADCY7 | na | 5559 | 0.149 | 0.1341 | Yes |
| 12 | TUBB3 | na | 5830 | 0.142 | 0.1423 | Yes |
| 13 | PDGFRA | na | 5971 | 0.138 | 0.1539 | Yes |
| 14 | SOS1 | na | 6194 | 0.132 | 0.1625 | Yes |
| 15 | HTR2C | na | 6768 | 0.117 | 0.1592 | Yes |
| 16 | PDGFA | na | 7165 | 0.108 | 0.1601 | Yes |
| 17 | TUBA8 | na | 7757 | 0.097 | 0.1540 | Yes |
| 18 | CDK1 | na | 7845 | 0.096 | 0.1623 | Yes |
| 19 | ADCY1 | na | 7855 | 0.095 | 0.1729 | Yes |
| 20 | GNAI3 | na | 7891 | 0.095 | 0.1826 | Yes |
| 21 | TUBA4A | na | 9187 | 0.072 | 0.1534 | No |
| 22 | PRKX | na | 9610 | 0.066 | 0.1487 | No |
| 23 | ITPR2 | na | 9655 | 0.065 | 0.1548 | No |
| 24 | SOS2 | na | 9955 | 0.061 | 0.1531 | No |
| 25 | PRKG2 | na | 10052 | 0.060 | 0.1570 | No |
| 26 | ITPR1 | na | 10204 | 0.058 | 0.1592 | No |
| 27 | PLCB3 | na | 10228 | 0.057 | 0.1650 | No |
| 28 | ADCY4 | na | 10290 | 0.056 | 0.1697 | No |
| 29 | ADCY6 | na | 10456 | 0.054 | 0.1711 | No |
| 30 | PRKCA | na | 10617 | 0.052 | 0.1724 | No |
| 31 | KRAS | na | 10630 | 0.052 | 0.1780 | No |
| 32 | EGFR | na | 11115 | 0.046 | 0.1692 | No |
| 33 | MAPK7 | na | 11510 | 0.041 | 0.1625 | No |
| 34 | TUBB6 | na | 12713 | 0.029 | 0.1312 | No |
| 35 | GNAI2 | na | 13045 | 0.027 | 0.1247 | No |
| 36 | TUBA3C | na | 13113 | 0.026 | 0.1257 | No |
| 37 | EGF | na | 13590 | 0.022 | 0.1145 | No |
| 38 | TUBB4B | na | 14185 | 0.017 | 0.0993 | No |
| 39 | MAP2K5 | na | 14281 | 0.017 | 0.0984 | No |
| 40 | TUBA1C | na | 15514 | 0.007 | 0.0637 | No |
| 41 | GNA11 | na | 15711 | 0.006 | 0.0587 | No |
| 42 | TJP1 | na | 15790 | 0.005 | 0.0571 | No |
| 43 | TUBA3D | na | 15967 | 0.004 | 0.0524 | No |
| 44 | GNAS | na | 16257 | 0.002 | 0.0443 | No |
| 45 | ADCY5 | na | 16504 | 0.000 | 0.0372 | No |
| 46 | MAP2K2 | na | 16997 | -0.003 | 0.0234 | No |
| 47 | PRKCG | na | 17545 | -0.007 | 0.0084 | No |
| 48 | GNAQ | na | 17828 | -0.009 | 0.0013 | No |
| 49 | ITPR3 | na | 18064 | -0.011 | -0.0043 | No |
| 50 | PLCB4 | na | 18133 | -0.011 | -0.0050 | No |
| 51 | HRAS | na | 18435 | -0.013 | -0.0121 | No |
| 52 | GJA1 | na | 18622 | -0.014 | -0.0159 | No |
| 53 | PRKACB | na | 18969 | -0.017 | -0.0240 | No |
| 54 | HTR2B | na | 19568 | -0.021 | -0.0389 | No |
| 55 | TUBA1B | na | 20167 | -0.025 | -0.0533 | No |
| 56 | TUBB | na | 20342 | -0.026 | -0.0554 | No |
| 57 | CSNK1D | na | 21052 | -0.032 | -0.0722 | No |
| 58 | GRM1 | na | 21083 | -0.032 | -0.0695 | No |
| 59 | ADRB1 | na | 21666 | -0.037 | -0.0821 | No |
| 60 | TUBA1A | na | 21769 | -0.037 | -0.0808 | No |
| 61 | MAPK3 | na | 22614 | -0.046 | -0.0999 | No |
| 62 | GRB2 | na | 23043 | -0.051 | -0.1066 | No |
| 63 | RAF1 | na | 23336 | -0.054 | -0.1089 | No |
| 64 | PRKACA | na | 23665 | -0.058 | -0.1118 | No |
| 65 | SRC | na | 24048 | -0.063 | -0.1156 | No |
| 66 | TUBB2A | na | 24361 | -0.067 | -0.1170 | No |
| 67 | MAPK1 | na | 24626 | -0.071 | -0.1166 | No |
| 68 | PRKACG | na | 25752 | -0.087 | -0.1393 | No |
| 69 | PDGFD | na | 25857 | -0.088 | -0.1323 | No |
| 70 | ADCY8 | na | 25957 | -0.090 | -0.1250 | No |
| 71 | MAP3K2 | na | 26106 | -0.092 | -0.1188 | No |
| 72 | DRD1 | na | 27274 | -0.114 | -0.1396 | No |
| 73 | GNAI1 | na | 27299 | -0.115 | -0.1273 | No |
| 74 | PDGFC | na | 27761 | -0.125 | -0.1264 | No |
| 75 | NRAS | na | 27790 | -0.126 | -0.1130 | No |
| 76 | DRD2 | na | 28135 | -0.135 | -0.1076 | No |
| 77 | HTR2A | na | 29146 | -0.161 | -0.1186 | No |
| 78 | PDGFRB | na | 29523 | -0.173 | -0.1098 | No |
| 79 | TUBB1 | na | 30796 | -0.214 | -0.1222 | No |
| 80 | MAP2K1 | na | 31070 | -0.223 | -0.1048 | No |
| 81 | GUCY1A2 | na | 31473 | -0.238 | -0.0894 | No |
| 82 | TUBB2B | na | 31922 | -0.257 | -0.0732 | No |
| 83 | PLCB1 | na | 32251 | -0.272 | -0.0518 | No |
| 84 | PDGFB | na | 32445 | -0.283 | -0.0252 | No |
| 85 | PRKCB | na | 33863 | -0.399 | -0.0209 | No |
| 86 | PRKG1 | na | 33894 | -0.404 | 0.0241 | No |
Table: GSEA details [plain text format]

  

Fig 2: KEGG\_GAP\_JUNCTION      
 Blue-Pink O' Gram in the Space of the Analyzed GeneSet

  

Fig 3: KEGG\_GAP\_JUNCTION: Random ES distribution      
 Gene set null distribution of ES for **KEGG\_GAP\_JUNCTION**
